# Effects of A Concentrate Rich in Agro-Industrial By-Products on Productivity Results, Carcass Characteristics and Meat Quality Traits of Finishing Heifers

**DOI:** 10.3390/ani10081311

**Published:** 2020-07-30

**Authors:** María José Moreno Díaz, Valeriano Domenech García, Carmen Avilés Ramírez, Francisco Peña Blanco, Francisco Requena Domenech, Andrés Luis Martínez Marín

**Affiliations:** Departamento de Producción Animal, Universidad de Córdoba, Ctra. Madrid-Cádiz, km 396. Campus de Rabanales, 14071 Córdoba, Spain; v22modim@uco.es (M.J.M.D.); pa1dogav@uco.es (V.D.G.); v92avrac@uco.es (C.A.R.); pa1peblf@uco.es (F.P.B.); v02redof@uco.es (F.R.D.)

**Keywords:** by-products, finishing heifers, performance, carcass, meat, ageing

## Abstract

**Simple Summary:**

In geographic areas where it is not possible to finish beef cattle on pastures or preserved forages, diets are mainly based on cereals. This intensive feeding system competes for feed with other species and does not take advantage of the capacity of ruminants to digest fibrous feeds. Moreover, cereal-rich diets for ruminants cannot be considered sustainable under current policy guidelines and make no significant contribution to the circular bioeconomy. We assayed an alternative intensive finishing diet for beef heifers where agro-industrial by-products, some of them highly fibrous ones, comprised 73.5% of the concentrate. We found that there were no differences in growth performance, carcass characteristics and meat quality traits between treatments, which might be considered positive from the point of view of sustainability of beef production. We also found that instrumental measures of meat related to important sensory attributes for consumer acceptance can be improved by increasing ageing time from 7 to 21 or 28 days.

**Abstract:**

Finishing diets in intensive beef production systems are mainly based on cereals, which does not take advantage of the capacity of the ruminant digestive system to digest fibrous feeds, cannot be considered sustainable and does not contribute to the circular bioeconomy. Our aim was to investigate the effects of an alternative concentrate rich in agro-industrial by-products for finishing crossbred Limousine heifers. Four pens with 12 heifers and four pens with 13 heifers were randomly allocated to one of two treatments: control (CON), a commercial concentrate with a 43.3% cereal composition, and alternative (ALT), a concentrate with a composition of 26% cereals and up to 73.5% agro-industrial by-products. Growth performance data were collected along the 91 days of the experimental period. Carcass characteristics were collected after slaughter and 24 h later. Vacuum-packaged samples from longissimus muscle were aged for 7, 21 or 28 days to study meat quality traits. Feed intake was higher and feed conversion rate was lower in the ALT treatment, but no differences were found in average daily gain and feeding costs. Treatment had no effects on any of the measured carcass traits (grading, hot and cold carcass weight, dressing out, chilling losses, subcutaneous fat depth, pH, temperature and lean and fat colour) nor on the meat quality traits (drip loss, cooking loss, shear force, oxidative stability, chromatic indices and pigment contents). Ageing time decreased drip loss and shear force, increased lightness and did not affect redness or surface colour stability. In conclusion, feeding crossbred Limousine heifers a finishing diet rich in agro-industrial by-products did not have any negative effects on performance, carcass and meat quality traits, which might be considered positive from the point of view of sustainability of beef production. Under the conditions assayed, ageing for 21 and 28 days improved tenderness of meat, without detrimental effects on oxidative stability or traits related to visual acceptability.

## 1. Introduction

The European Union is the world’s third largest producer of beef, but there are great differences in productivity and incomes of beef producers across regions due to diversity of climates, forage availability, breeds and livestock farming practices [1]. In Mediterranean areas, such as Southern Spain, it is not usually feasible to finish beef cattle on pastures or preserved forages due to climatic conditions. Therefore, after weaning at 5–8 months, calves are reared under intensive feedlot conditions where they are finished with ad libitum feeding of concentrates, mainly based on cereals, and limited consumption of cereal straw as a roughage source. These type of finishing diets may increase farm production costs, compete for feed with swine and poultry and do not take advantage of the capacity of ruminants to digest fibrous feeds [2]. Such intensive feeding systems can not be considered as sustainable under current policy guidelines, nor do they make any significant contribution to the circular bioeconomy [3,4]. An option to change the current situation could be to replace the cereals in the concentrate with agro-industrial by-products [5].

The nature of the finishing diet might influence productivity results, carcass traits and instrumental meat quality [6]. Replacing cereals with fibrous by-products usually decreases the energy content of the concentrate and might affect fat synthesis and deposition [7,8]. Finishing diet characteristics as well as length and conditions of meat storage and retail display can impact meat colour and tenderness [9], which is important since colour is used by consumers as an indicator of freshness and has a great impact on purchasing decision, whereas tenderness is the most appreciated feature when eating meat [10]. Some studies have described the responses of those traits after replacing part of the concentrate with silage in total mixed rations fed ad libitum in intensive feeding systems [11,12,13,14]. However, very few studies have dealt with productivity, carcass and meat traits of beef cattle finished indoors and fed high-concentrate diets where agro-industrial by-products are the main ingredients [15].

In the current research, finishing heifers reared in an intensive feedlot were fed a commercial concentrate or an alternative concentrate very rich in agro-industrial by-products, some of them highly fibrous ones. Our aim was to investigate the effects of the concentrate composition on growth performance, carcass characteristics and meat quality traits.

## 2. Material and Methods

### 2.1. Animals, Housing and General Management

All the experimental procedures used in the present study followed Spanish regulations for the care of animals during farming, transport, slaughter and experimentation and the European Union directive for animal experiments [16,17].

In a commercial intensive feedlot, one hundred Limousine crossbred heifers (50 per treatment), 12 ± 1.8 months old and with an initial body weight (BW) of 378 ± 10.3 kg, were allocated to eight pens (four pens with 12 heifers and four pens with 13 heifers), while ensuring uniformity in age and body weight among pens. The pens had a roofed straw-bedded area in front of the feed bunk and an outdoor area. Each pen had its own water trough and straw rack and the feed bunk was long enough to allow all animals to eat at the same time. Animals had free access to water, concentrate and cereal straw at any time. The experimental period lasted 91 days and was preceded by an adaptation period of 14 days, in which the animals were gradually changed from the growing diet (a commercial cereal-based concentrate plus cereal straw), common to all of them, to the appropriate finishing diet, according to the experimental design.

### 2.2. Experimental Design and Diets

The pens were randomly allocated to one of two treatments (four pens per treatment), namely control (CON) and alternative (ALT). The treatments only differed in the type of steam-pelleted concentrate that was fed to the animals (Table 1). The concentrate in the CON treatment was a typical commercial one for finishing heifers, mainly cereal-based. The ALT concentrate was composed of 26% cereals and up to 73.5% of agro-industrial by-products, some of them highly fibrous (Table 1). Both concentrates were fed ad libitum, and cereal straw was fed as the sole roughage source.

### 2.3. Measurements, Sample Collection and Laboratory Analyses

Samples of the diets were taken from each batch mix, and aliquots were pooled in one sample per concentrate for chemical analyses [19,20].

Average BW per pen was recorded 2 h before morning feed delivery at the beginning of the trial and every 30 days thereafter. Total feed intake in each pen was calculated as the sum of feed consumed throughout the experimental period. Following, average daily gain (ADG, kg/day), average feed intake (FI, kg/day) and feed conversion ratio (FCR, feed to gain kg/kg) were calculated for each pen.

On day 91 of the trial, all heifers were weighed, and three heifers per pen (12 heifers from each treatment) having the final BW closest to the median weight of the pen were tagged to track their carcasses. Then, the heifers were transported from the farm to a commercial abattoir located approximately 145 km (~1 h 40 min), in an adequately conditioned vehicle. After a 10-h rest period, the heifers were slaughtered by captive-bolt pistol, exsanguinated and dressed using standard commercial procedures. Thus, the carcasses were not electrically stimulated.

The carcasses were weighed (hot carcass weight (HCW)) and graded for conformation (E, excellent; U, very good, R, good; O, fair; P, poor) and fatness (1, low; 2, slight; 3, average; 4, high; 5, very high) by trained personnel at the abattoir, according to the EU official classification system for beef carcasses [21]. Afterwards, in the first 45 min after slaughter, temperature (Tª_0_), pH (pH_0_) and lean and subcutaneous fat colour of the carcasses were registered. Temperature and pH were monitored in the longissimus muscle by using a portable pH-meter (Crison PH25, Hach Lange, Barcelona, Spain) equipped with a glass electrode suitable for meat penetration and automatic temperature compensator. The probe was inserted in a scalpel incision approximately 1 cm into its geometrical centre at right angles to the sagittal plane surface. The pH electrode was recalibrated at room temperature every five samples, using two standard buffer solutions at pH 4.0 and 7.0, and rinsed between measurements. Three measurements for lean (rectus abdominis muscle) and caudal subcutaneous fat colours were taken with a hand-held spectrophotometer (CM-2600d, Minolta Co., Osaka, Japan; λ: 360–740, Δλ: 10 nm, specular component excluded mode, D65 standard illuminant, 10° visual angle and 8 mm measurement aperture), standardised against a white tile (L* = 97.78, a* = 0.19, b* = 1.84) and light trap supplied by the manufacturer. Colour coordinates were expressed as L* (lightness), a* (redness) and b* (yellowness), and the average value for each of them was reported and used for calculation of C* (chroma or vividness of h*) and h* (hue angle or the degree to which a colour stimulus can be described as red, green, yellow, blue or the combinations between them) as C* = (a*^2^ + b*^2^)^0.5^ and h* = arctangent (b*/a*) × 360°/(2 × π) [22]. For fat colour, readings were taken on subcutaneous fat covered with plastic food wrap (calibration was performed using the food wrap to maintain the integrity of the results). The carcasses were then split along the spine into two halves and chilled at 4 °C for 24 h in a commercial chiller. After, the half carcasses were reweighed to obtain the cold carcass weight (CCW), and temperature (Tª_24_) and pH (pH_24_) were measured as indicated above. The dressing was calculated as the ratio of cold carcass weight (CCW) to final BW at the farm, while chilling losses were calculated as the ratio of the difference between HCW and CCW to HCW. Both data were expressed as percentages.

The longissimus muscle was cut out from the fifth to the last thoracic vertebrae of the chilled left half carcasses and cut into three portions that were individually packed in sealed vacuum bags to get contact between the bag and the meat. The bags were identified and delivered via refrigerated transport (4 °C) to the laboratory. At the laboratory, dorsal fat thickness was measured with a stainless steel calliper at the 12th to 13th rib interfaces, over the longissimus muscle at a point three-quarters the ventral length of the ribeye (FT1) and over the latissimus dorsi muscle (FT2). The portion in each bag within each animal of origin was considered a sample that was randomly assigned to one of three different ageing times (7, 21 and 28 days) at 2–4 °C in darkness on stainless steel gratings. The bags were turned over and rotated among shelf positions every day to minimise location effects. After each ageing time, meat samples were unpacked, and pH was immediately determined. Meat samples were then subsampled as needed for determinations of drip loss (DL), cooking loss (CL), Warner–Bratzler shear force (WBSF), colour, pigment contents and oxidative stability.

For DL measurement, a block of meat trimmed of external fat and connective tissue and measuring 20 × 20 × 25 mm, was sliced so that the fibres ran across the longer axis of the sample. The slices were weighed and suspended on metal hooks placed on the inner side of the lid of a plastic airtight container so that the sample did not touch the container walls. After display for 24, 48 and 72 h at 4 °C, the slices were carefully dabbed and weighed again. The DL of each slice was calculated as the percent weight difference between the initial and final weight relative to the initial weight.

To assess CL and WBSF, a steak of 5 cm length was cut from the cranial end of each sample. Steaks were trimmed of external fat and epimysium and weighed prior to cooking. The steaks were individually placed inside plastic bags and boiled using a water bath (Precisterm 6000388, J.P. Selecta Co., Barcelona, Spain), which was preheated to 75 °C, to a final internal temperature of 71 °C [23]. Internal temperature was monitored by an iron/constant thermocouple wire connected to a thermometer (HI 98,509 Checktemp Pocket Thermometer, Hanna Instruments, Guipúzcoa, Spain) inserted into the geometric centre of the steak. After cooking, the steaks were cooled at room temperature for 30 min, gently blotted dry by using paper towels and weighed again. Cooking loss (CL) was determined by calculating the weight difference of the steaks before and after cooking, expressed as percentage of initial weight. Then, five 1.27 cm diameter cores were removed parallel to the muscle fibre orientation from the lateral end of the cooked steaks. Peak WBSF (kg/cm^2^) was measured perpendicular to the muscle fibres using a TA.XT-2 texture analyser (Texture Analyser, Stable Micro Systems, Surrey, UK) equipped with a Warner–Bratzler shear device (25 kg load cell) and a crosshead speed of 200 mm/min. The down stroke distance was 3 cm (the probe should cut the meat completely). Each core was assessed two times, and the 10 peak shear forces recorded per sample were averaged for statistical analysis.

For meat colour analysis, the steaks were placed on styrofoam trays, overwrapped with oxygen-permeable PVC film (O_2_ permeability = 15,500–16,200 cm^3^/m^2^/24 h at 23 °C) and placed in a dark chill room at 4 °C for 30 min to permit blooming before L*, a* and b* values were recorded (day 0 of colour measurement). Three measurements, changing the spectrophotometer orientation at each time, were made directly on the meat surface immediately after removing the film at non-overlapped zones of each steak, which avoided areas of connective tissue and intramuscular fat. The three values of L*, a* and b* were averaged for statistical analysis and C* and h* values were derived. Then, the samples were overwrapped again and kept in the dark at 4 °C for further colour analysis after 24 h of aerobic display. Overall colour variation between the initial measurement when removed from the vacuum package (0) and the next measurement at display (24) was determined using the colour difference coefficient (ΔE), which was calculated as ΔE = ((L*_0_ − L*_24_)^2^ + (a*_0_ − a*_24_)^2^ + (b*_0_ − b*_24_)^2^)^0.5^. Additionally, spectral data were used to estimate the surface colour stability (SCE) as R630/R580 [24]. The reflectance values at four wavelengths (473, 525, 572 and 700 nm) were used to calculate the proportions of metmyoglobin as MMb = 2.375 × [1 − ((A473 − A700)/(A525 − A700))] × 100, deoxymyoglobin as DMb = 1.395 − ((A572 − A700)/(A525 − A700)) × 100 and oxymyoglobin as OMb = 100 − (AD + AM), where A473, A525, A572 and A700 are the common logarithms of the reciprocals of reflectance values at 473, 525, 572 and 700 nm, respectively [25].

The extent of lipid oxidation at each ageing time was assessed by measuring thiobarbituric acid reactive substances (TBARS), expressed as mg of malondialdehyde (MDA)/kg of meat [26].

### 2.4. Statistical Analyses

SAS UE 3.8 (SAS Institute Inc., Cary, NC) was used to perform the statistical analyses. Statistical significance was declared at *p* < 0.05. All quantitative data were checked for normality before analyses by means of Kolmogorov–Smirnov test. Productivity data were analysed with the GLM procedure, using the pen (12–13 animals per pen) as experimental unit and the treatment as fixed effect. Carcass traits, except for grading, were analysed with the MIXED procedure. The statistical model included the treatment as fixed effect and the pen (four pens per treatment and three samples per pen) nested within treatment as random effect. The pH_0_ was used as covariate in the analysis of lean muscle colour [27]. Carcass grading data were submitted to a chi-squared test with the FREQ procedure. A repeated measurements analysis of meat data was carried out with the MIXED procedure. A covariance structure appropriate for unequally spaced measures (compound symmetry, ANTE(1) or spatial power) was chosen for each variable on the basis of the Schwarz’s Bayesian information model fit criteria. The statistical model included the fixed effects of treatment, ageing time and their interaction; the repeated effect was ageing time; the subject of the repeated measurements was the animal nested within treatment and pen; and the pH at the end of each ageing time was used as covariate in the analyses of DL, CL, WBSF, colour traits and pigment contents. When the fixed effects of the repeated measurement model were significant, differences between least squares means were assessed by paired t-test. Pearson’s correlation was used when appropriate.

## 3. Results

Except for FI and FCR, no differences (*p* > 0.05) were found in the productivity results between treatments (Table 2). The ALT concentrate was about 12% cheaper than the CON concentrate (169.13 vs. 191.88 €/t, calculated using local market prices of feedstuffs in the year 2017), but no differences were observed in the feeding cost per heifer in the 91-day period or in the cost per kg of body weight gain, since the higher FI in the ALT treatment fully compensated for the lower price of its concentrate, at nearly the same ADG in both treatments.

Treatment did not affect (*p* > 0.05) carcass characteristics (Table 3) or meat traits (Table 4). However, ageing time affected (*p* < 0.05) pH, DL, WBSF, TBARS, chromatic indices (except for a*, C* and SCE) and pigment contents of meat. The pH showed a quadratic pattern of change with a maximum at 21 days of ageing. The DL and WBSF decreased between 7 and 21 days of ageing time and stabilised thereafter at average values of 0.76% and 5.05 kg/cm^2^, respectively. The TBARS linearly increased with ageing to a maximum of 0.67 mg MDA/kg. The L* values were higher at 21 and 28 than at 7 days of ageing. The b* and h* values showed a quadratic pattern of change with a minimum at 21 days of ageing, while, on the contrary, the ΔE values were higher at 21 days than at 7 or 28 days of ageing. The changes of MMb and DMb with ageing were positive linear and quadratic with a maximum at 21 days, respectively. The OMb values decreased with ageing time and were higher at 7 days than at 21 and 28 days of ageing. The CL values did not reach significant differences but showed a decreasing linear trend (*p* = 0.06).

## 4. Discussion

The dietary treatments tested in the present work differed in the composition of the pelleted concentrates that were used in each of them (Table 1). The concentrate in the CON diet was mainly based on cereals and included 31.4% by weight of agro-industrial by-products. However, the concentrate in the ALT diet was mainly based on agro-industrial by-products, which sum up to 73.5% by weight of the concentrate. Some by-products were common in both concentrates, but the ALT concentrate was the only one that included highly fibrous by-products, like soybean hulls, NaOH-treated wheat straw, camelina husks and extracted grape seed meal, which represented up to 24% by weight of the concentrate, i.e., they were present in greater amount than cereals. As a result of the differences in ingredient composition, the NDF-to-starch ratio was higher and the net energy content was lower in the ALT treatment as compared to the CON treatment (0.9 vs. 0.6 and 5.7 vs. 6.4 MJ net energy for maintenance and weight gain/kg of feed as fed). Therefore, effects on the productivity results, carcass characteristics and meat quality traits, if any, could be only ascribed to such differences.

The lack of differences in productivity results between treatments observed in the present work (Table 2) agreed well with previous research where the diets assayed had larger differences in the starch contents (10 to 20 percentage points between diets) than that in the present work [13,14,15]. Moreover, other authors did not find differences between diets with NDF contents that were in the same range as those in our work [11,12].

The fact that the heifers in the ALT treatment had the same ADG and higher FI than those in the CON treatment suggested that growth potential, and its energy demand added to that for body maintenance, drove the FI in order to maximise energy intake, which is supported by the similar consumption of net energy for maintenance and weight gain in both treatments (47.9 vs. 48.6 MJ/d in the CON and ALT treatments, respectively; from Table 1 and Table 2). Moreover, meta-analysis of published research showed that the NDF content in feedlot cattle diets (within a range of 7.5 to 35.3%) was positively related to FI with no effects on net energy for weight gain per kg of dry matter intake [28]. Again, the ADG values in the present study were lower than those recorded in Limousine bulls at a similar energy intake [11], which may be ascribed to the well-known lower weight gain capacity of heifers compared with bulls [29].

The absence of differences in the carcass characteristics between treatments (Table 3) agreed with the results reported by other authors [12,14,15]. However, a higher carcass fatness score and FT2, and consequently greater Tª_0_ due to higher carcass insulation, were observed when a low-concentrate diet was fed to bulls [11], while heifers fed a high-concentrate diet showed a higher carcass fatness score than those fed a low-concentrate diet [13]. From our results and those from previous studies [11,13,14,15], no clear relationship could be established between the carbohydrate composition of indoor finishing diets (i.e., the relative proportions of substrates for fatty acid synthesis in the tissues) and subcutaneous fat deposition, as previously suggested [8]. Again, C* and a* values of rectus abdominis muscle measured in the first 45 min postmortem were found to be predictors of colour stability (expressed as MMb) after blooming for 48 h, depending on ageing time (C* after 3 and 14 days of ageing and a* after 7 days of ageing) [30]. However, in our study, we did not find any significant correlations between the C* or a* values of rectus abdominis muscle and MMb at any ageing time after 24 of aerobic display.

The pattern of change of pH (Table 4) coincided with previous observations [31]. All the pH values in our study were within the intermediate range (5.80–6.19) that is related to meat samples with the lowest observed tenderization [32].

The DL decreased between 7 and 21 days of ageing (Table 4), which agreed with the increasing capacity of water retention of meat throughout ageing. Water loss of meat during storage is related to how much interfibrillar water is released to the cytoplasm and how easily it can leave the muscle cells and muscle fibre bundles through the so-called drip channels [33]. Ageing increases water holding capacity, probably by disrupting those drip channels [34]. Since water holding capacity of fresh meat is important for visual acceptability, the most appropriate ageing time of the meat obtained under the conditions assayed in the present work would be at least 21 days.

In line with our results (Table 4), other authors reported no differences in CL between their treatments and values of around 25% without a significant effect of ageing time [11]. On the contrary, a higher CL in the meat from young bulls fed a finishing sugar-beet pulp diet was reported [15]. The decreasing linear trend of CL with ageing time (Table 4) was unexpected and might be related to the pattern of degradation of myofibrillar, cytoskeletal and sarcoplasmic proteins during ageing [35,36]. The observed CL values at any ageing time would allow the meat to be classified as intermediate juicy by consumers [37,38].

The lack of differences in WBSF between treatments (Table 4) disagreed with previous findings [15]. Furthermore, the observed pattern of change of WBSF concurred with the findings of some authors but not others [11,39]. Taking into account the average WBSF at each ageing time, from a sensory point of view, our cooked meat samples would be perceived by consumers as tough at 7 days of ageing (6.7 ± 1.13 kg/cm^2^) and only as intermediate tender after 21 and 28 days of ageing (5.3 ± 0.89 and 4.1 ± 0.71 kg/cm^2^, respectively [40]. Therefore, under the conditions assayed in the present work, meat would require at least 21 days of ageing to obtain a product with acceptable sensory tenderness, without loss of juiciness, after cooking. The WBSF at any ageing time was not correlated with the lean muscle colour values measured in the first 45 min after slaughtering in coincidence with other authors [41].

The TBARS increase with ageing time (Table 4) concurred with previous observations [42]. The TBARS values in our study were well below 2, which can be considered the limiting threshold for the acceptability of oxidised beef [43]. In agreement with previous research, TBARS were negatively correlated with a* (r = −0.32; *p* < 0.01) [44].

Other authors found that a* decreases and MMb increases in vacuum-packaged meat with longer ageing [42], which only partially agreed with our results (Table 4). Moreover, our results totally disagreed with the absence of differences in L*, b* and DMb due to ageing reported in previous research [45]. All meat samples reached the threshold value for a* of 14.5 to be considered visually acceptable for consumers [46].

The ΔE of meat (Table 4) has not received enough attention in meat colour research, taking into account that it measures the combined effects of L*, a* and b* changes on total colour changes [47]. According to these authors, a*, C* and SCE are negatively correlated while ΔE is positively correlated with MMb. Besides, ΔE and SCE are even more sensitive to changes in MMb than a* or C*. We found a negative correlation between ΔE and MMb (r = −0.27; *p* < 0.05), but we observed no correlation of a*, C* and SCE with MMb.

The SCE is used as an indicator for redness reduction and brownness increase on a meat surface (Table 4). We did not observe any effect of ageing time on SCE, which is contrary to previous findings [48]. Translating the SCE threshold value of 3.3 for consumer acceptance of lamb [49] to beef, all meat samples in the present study were acceptable irrespective of ageing time.

## 5. Conclusions

Feeding crossbred Limousine heifers a finishing diet rich in agro-industrial by-products, mostly fibrous ones, has no negative effects on productivity, carcass and meat quality traits, which might be considered positive from the point of view of beef production sustainability. Under the conditions assayed, ageing for at least 21 days allowed us to obtain meat of acceptable shear force and better water holding capacity as compared with ageing for 7 days, without worsening cooking loss, lightness, redness or oxidative stability.

## Figures and Tables

**Table 1:** Ingredients and analysed composition of experimental concentrates (control (CON): conventional concentrate; alternative (ALT): concentrate rich in agro-industrial by-products).

	CON	ALT
Barley (%)	32.3	16.0
Maize	11.0	6.0
Agro-industrial by-products (%)	31.4 ^†^	73.5 ^‡^
Sunflower meal (%)	8.0	-
Bitter vetch (%)	5.0	-
Palm kernel meal (%)	4.0	-
Soybean meal (%)	2.0	-
Calcium salts of palm fatty acids (%)	1.1	-
Palm oil (%)	0.7	-
Minerals and vitamins (%)	4.5	4.5
Chemical composition, as fed		
Dry matter (%)	89.8	89.4
Crude protein (%)	14.0	14.0
Fat by acid hydrolysis (%)	4.7	4.6
Neutral detergent fibre (%)	21.9	27.4
Acid detergent fibre (%)	10.4	16.4
Lignin (%)	1.8	3.0
Starch (%)	34.5	29.4
Ash (%)	7.6	7.5
NEmg (MJ/kg as fed)	6.4	5.7

^†^ Corn gluten feed (9.0%), wheat bran (8.0%), hominy feed (4.0%), corn dried distiller grains with solubles (4.0%), dehydrated barley sprouts (4.0%) and rice bran (2.4%). ^‡^ Soybean hulls (12.0%), hominy feed (11.2%), corn dried distiller grains with solubles (10.0%), wheat bran (8.2%), corn gluten feed (8.1%), dehydrated barley sprouts (6.0%), NaOH-treated wheat straw (5.0%), camelina meal (4.0%), camelina husks (4.0%), extracted grape seed meal (3.0%) and rice bran (2.0%). NEmg: net energy for maintenance and weight gain [18].

**Table 2:** Productivity results of crossbred Limousine heifers finished on a conventional concentrate (CON) or a concentrate rich in agro-industrial by-products (ALT).

Parameters	Diet		SEM	*p*
	CON	ALT		
Initial body weight (kg)	379	379	2.1	0.92
Final body weight (kg)	469	470	3.0	0.88
Average daily gain (kg/day)	0.99	1.01	0.018	0.65
Average feed intake (kg/day)	7.48	8.53	0.214	<0.001
Feed conversion rate (kg/kg)	7.60	8.48	0.199	0.01
Feeding cost (€/animal/period)	130.6	131.2	1.34	0.83
Fattening cost (€/kg body weight gain)	1.46	1.43	0.021	0.63

SEM: standard error of the mean.

**Table 3:** Carcass characteristics of crossbred Limousine heifers finished on a conventional concentrate (CON) or a concentrate rich in agro-industrial by-products (ALT).

Traits	Diet		SEM	*p*
	CON	ALT		
Grading	58% R3/42% U3	33% R3/67% U3	-	0.22
Hot carcass weight (kg)	259	266	2.3	0.19
Cold carcass weight (kg)	251	264	2.2	0.19
Dressing out ^1^ (%)	55.4	56.1	0.43	0.45
Chilling losses ^2^ (%)	1.99	2.00	0.002	0.12
FT1 (cm)	3.76	5.23	0.042	0.14
FT2 (cm)	7.44	9.32	0.052	0.07
pH_0_	6.62	6.72	0.034	0.17
pH_24_	5.97	5.79	0.071	0.27
Tª_0_ (°C)	40.2	39.6	0.18	0.12
Tª_24_ (°C)	2.9	3.1	0.08	0.18
Muscle				
L*	35.4	33.9	0.97	0.58
a*	14.3	16.7	0.68	0.31
b*	12.2	13.3	0.42	0.19
C*	19.2	21.2	0.675	0.29
h*	40.9	38.9	1.184	0.58
Fat				
L*	83.4	86.8	0.96	0.16
a*	0.44	1.00	0.192	0.27
b*	9.43	9.99	0.402	0.50
c*	9.47	10.10	0.400	0.46
h*	85.1	82.5	0.765	0.70

^1^ Calculated as (hot carcass weight/final body weight) × 100. ^2^ Calculated as 100 – ((cold carcass weight/hot carcass weight) × 100). SEM: standard error of the mean; FT1: fat thickness over longissimus muscle; FT2: fat thickness over latissimus dorsi muscle; L*: lightness; a*: redness; b*: yellowness; C*: chroma; h*: hue.

**Table 4:** Values of pH, drip loss (DL), cooking loss (CL, %), Warner–Braztler shear force (WBSF, kg/cm^2^), thiobarbituric acid reactive substances (TBARS, mg malonaldehyde/kg meat), colour traits (L*, lightness; a*, redness; b*, yellowness; C*, chroma; h*, hue), colour variation (ΔE), surface colour stability (SCE) and pigment contents (MMb, metmyoglobin; DMb, deoxymyoglobin; OMb, oxymyoglobin; all in % of total pigments) of meat from crossbred Limousine heifers finished on a conventional concentrate (CON) or a concentrate rich in agro-industrial by-products (ALT) and vacuum-aged for 7, 21 or 28 days.

Traits	Diet (D)						SEM	*p*		
	CON			ALT						
	Ageing Time (A)			Ageing Time						
	7	21	28	7	21	28		D	A	D × A
pH	5.90 ^b^	6.00 ^a^	5.90 ^b^	5.92	5.99	5.96	0.012	0.47	<0.01	0.47
DL	1.57 ^a^	0.78 ^b^	0.78 ^b^	1.38 ^a^	0.69 ^b^	0.78 ^b^	0.057	0.30	<0.001	0.64
CL	26.1	25.5	25.1	25.7	25.3	23.6	0.26	0.21	0.06	0.55
WBSF	6.63 ^a^	5.58 ^b^	4.99 ^b^	6.69 ^a^	5.00 ^b^	4.62 ^b^	0.143	0.38	<0.001	0.45
TBARS	0.58 ^b^	0.59 ^ab^	0.67 ^a^	0.56 ^b^	0.60 ^ab^	0.67 ^a^	0.012	0.89	<0.01	0.77
L*	34.8 ^c^	39.0 ^a^	37.1 ^b^	35.5 ^b^	38.2 ^a^	37.7 ^a^	0.33	0.77	<0.001	0.53
a*	14.4	14.9	14.6	14.7	14.6	15.0	0.21	0.78	0.86	0.77
b*	22.1 ^a^	16.4 ^c^	19.7 ^b^	21.3 ^a^	16.0 ^b^	20.4 ^a^	0.33	0.71	<0.001	0.16
C*	22.8	22.2	23.4	23.4	21.7	22.4	0.27	0.44	0.66	0.26
h*	51.7 ^a^	48.0 ^b^	51.3 ^a^	51.4 ^a^	48.2 ^b^	51.7 ^a^	0.40	0.89	<0.001	0.93
MMb	8.80 ^b^	10.4 ^b^	13.3 ^a^	8.81 ^b^	10.4 ^b^	13.0 ^a^	0.323	0.88	<0.001	0.96
DMb	14.8 ^c^	24.6 ^a^	21.3 ^b^	15.7 ^c^	27.6 ^a^	19.7 ^b^	0.76	0.48	<0.001	0.23
OMb	76.4 ^a^	65.0 ^b^	65.4 ^b^	75.5 ^a^	61.9 ^c^	67.3 ^b^	0.89	0.58	<0.001	0.29
ΔE	9.04 ^b^	16.1 ^a^	5.71 ^c^	7.82 ^b^	16.6 ^a^	5.74 ^c^	0.566	0.52	<0.001	0.18
SCE	4.24	4.45	4.31	4.30	4.57	4.37	0.073	0.62	0.38	0.99

^a,b^ For each variable, within each diet, least squares means without a common superscript letter are different by t-test at *p* < 0.05. SEM: standard error of the mean.

## References

[1] Hocquette J.F., Ellies-Oury M.P., Lherm M., Pineau C., Deblitz C., Farmer L. (2018). Current situation and future prospects for beef production in Europe–A review. Asian-Australas. J. Anim. Sci..

[2] Mottet A., Teillard F., Boettcher P., De Besi G., Besbes B. (2018). Review: Domestic herbivores and food security: Current contribution, trends and challenges for a sustainable development. Animal.

[3] FAO (2010). Sustainable Diets and Biodiversity: Directions and Solutions for Policy, Research and Action.

[4] Van Zanten H.H.E., Van Ittersum M.K., De Boer I.J.M. (2019). The role of farm animals in a circular food system. Glob. Food Sec..

[5] Salami S.A., Luciano G., O’Grady M.N., Biondi L., Newbold C.J., Kerry J.P., Priolo A. (2019). Sustainability of feeding plant by-products: A review of the implications for ruminant meat production. Anim. Feed Sci. Technol..

[6] Mwangi F.W., Charmley E., Gardiner C.P., Malau-Aduli B.S., Kinobe R.T., Malau-Aduli A.E.O. (2019). Diet and genetics influence beef cattle performance and meat quality characteristics. Foods.

[7] Ludden P.A., Cecava M.J., Hendrix K.S. (1995). The value of soybean hulls as a replacement for corn in beef cattle diets formulated with or without added fat. J. Anim. Sci..

[8] Rhoades R.D., Sawyer J.E., Chung K.Y., Schell M.L., Lunt D.K., Smith S.B. (2007). Effect of dietary energy source on in vitro substrate utilization and insulin sensitivity of muscle and adipose tissues of Angus and Wagyu steers. J. Anim. Sci..

[9] López-Pedrouso M., Rodríguez-Vázquez R., Purriños L., Oliván M., García-Torres S., Sentandreu M.Á., Lorenzo J.M., Zapata C., Franco D. (2020). Sensory and physicochemical analysis of meat from bovine breeds in different livestock production systems, pre-slaughter handling conditions, and ageing time. Foods.

[10] Glitsch K. (2000). Consumer perceptions of fresh meat quality: Cross-national comparison. Br. Food J..

[11] Avilés C., Martínez A.L., Domenech V., Peña F. (2015). Effect of feeding system and breed on growth performance, and carcass and meat quality traits in two continental beef breeds. Meat Sci..

[12] Bodas R., Posado R., Bartolomé D.J., de Paz M.J.T., Herráiz P., Rebollo E., Gómez L.J., García J.J. (2014). Ruminal pH and temperature, Papilla characteristics, and animal performance of fattening calves fed concentrate or maize silage-based diets. Chil. J. Agric. Res..

[13] Casasús I., Ripoll G., Albertí P. (2012). Use of maize silage in beef heifers fattening diets: Effects on performance, carcass and meat quality. ITEA.

[14] Cooke D.W.I., Monahan F.J., Brophy P.O., Boland M.P. (2004). Comparison of concentrates or concentrates plus forages in a total mixed ration or discrete ingredient format: Effects on beef production parameters and on beef composition, colour, texture and fatty acid profile. Ir. J. Agric. Food Res..

[15] Cuvelier C., Cabaraux J.F., Dufrasne I., Clinquart A., Hocquette J.-F., Istasse L., Hornick J.L. (2006). Performance, slaughter characteristics and meat quality of young bulls from Belgian Blue, Limousin and Aberdeen Angus breeds fattened with a sugar-beet pulp or a cereal-based diet. Anim. Sci..

[16] BOE (2013). Ley 6/2013, de 11 de junio, de modificación de la Ley 32/2007, de 7 de noviembre, para el cuidado de los animales, en su explotación, transporte, experimentación y sacrificio. BOE.

[17] EC (2010). Directive 2010/63/EU of the European Parliament and of the Council of 22 September 2010 on the protection of animals used for scientific purposes. Off. J..

[18] INRA (2007). Alimentation des Bovins, Ovins et Caprins.

[19] AOAC (2000). Official Methods of Analysis.

[20] Van Soest P.J., Robertson J.B., Lewis B.A. (1991). Methods for dietary fiber, neutral detergent fiber, and nonstarch polysaccharides in relation to animal nutrition. J. Dairy Sci..

[21] EC (2006). Council Regulation (EC) No 1183/2006 of 24 July 2006 concerning the Community scale for the classification of carcasses of adult bovine animals. Off. J..

[22] CIE (1986). Colorimetry.

[23] AMSA (2015). Research Guidelines for Cookery, Sensory Evaluation and Instrumental Tenderness Measurements of Meat.

[24] Hunt M.C. Meat Color Measurements. Proceedings of the 33rd Annual Reciprocal Meat Conference.

[25] AMSA (2012). Meat Color Measurement Guidelines.

[26] Tarladgis B.G., Watts B.M., Younathan M.T., Dugan L. (1960). A distillation method for the quantitative determination of malonaldehyde in rancid foods. J. Am. Oil Chem. Soc..

[27] Page J.K., Wulf D.M., Schwotzer T.R. (2001). A survey of beef muscle color and pH. J. Anim. Sci..

[28] Arelovich H.M., Abney C.S., Vizcarra J.A., Galyean M.L. (2008). Effects of dietary neutral detergent fiber on intakes of dry matter and net energy by dairy and beef cattle: Analysis of published data. Prof. Anim. Sci..

[29] Bittante G., Cecchinato A., Tagliapietra F., Verdiglione R., Simonetto A., Schiavon S. (2018). Crossbred young bulls and heifers sired by double-muscled Piemontese or Belgian Blue bulls exhibit different effects of sexual dimorphism on fattening performance and muscularity but not on meat quality traits. Meat Sci..

[30] Beriain M.J., Goñi M.V., Indurain G., Sarriés M.V., Insausti K. (2009). Predicting Longissimus dorsi myoglobin oxidation in aged beef based on early post-mortem colour measurements on the carcass as a colour stability index. Meat Sci..

[31] Wyrwisz J., Moczkowska M., Kurek M., Stelmasiak A., Półtorak A., Wierzbicka A. (2016). Influence of 21 days of vacuum-aging on color, bloom development, and WBSF of beef semimembranosus. Meat Sci..

[32] Lomiwes D., Farouk M.M., Wu G., Young O.A. (2014). The development of meat tenderness is likely to be compartmentalised by ultimate pH. Meat Sci..

[33] Hughes J.M., Oiseth S.K., Purslow P.P., Warner R.D. (2014). A structural approach to understanding the interactions between colour, water-holding capacity and tenderness. Meat Sci..

[34] Farouk M.M., Mustafa N.M., Wu G., Krsinic G. (2012). The “sponge effect” hypothesis: An alternative explanation of the improvement in the waterholding capacity of meat with ageing. Meat Sci..

[35] Boakye K., Mittal G.S. (1993). Changes in pH and water holding properties of Longissimus dorsi muscle during beef ageing. Meat Sci..

[36] Purslow P.P., Oiseth S., Hughes J., Warner R.D. (2016). The structural basis of cooking loss in beef: Variations with temperature and ageing. Food Res. Int..

[37] Lucherk L.W., O’Quinn T.G., Legako J.F., Rathmann R.J., Brooks J.C., Miller M.F. (2016). Consumer and trained panel evaluation of beef strip steaks of varying marbling and enhancement levels cooked to three degrees of doneness. Meat Sci..

[38] O’Quinn T.G., Legako J.F., Brooks J.C., Miller M.F. (2018). Evaluation of the contribution of tenderness, juiciness, and flavor to the overall consumer beef eating experience. Transl. Anim. Sci..

[39] Wu G., Farouk M.M., Clerens S., Rosenvold K. (2014). Effect of beef ultimate pH and large structural protein changes with aging on meat tenderness. Meat Sci..

[40] Destefanis G., Brugiapaglia A., Barge M.T., Dal Molin E. (2008). Relationship between beef consumer tenderness perception and Warner-Bratzler shear force. Meat Sci..

[41] Goñi M.V., Beriain M.J., Indurain G., Insausti K. (2007). Predicting longissimus dorsi texture characteristics in beef based on early post-mortem colour measurements. Meat Sci..

[42] Mancini R.A., Ramanathan R. (2014). Effects of postmortem storage time on color and mitochondria in beef. Meat Sci..

[43] Campo M.M., Nute G.R., Hughes S.I., Enser M., Wood J.D., Richardson R.I. (2006). Flavour perception of oxidation in beef. Meat Sci..

[44] Insausti K., Beriain M.J., Lizaso G., Carr T.R., Purroy A. (2008). Multivariate study of different beef quality traits from local Spanish cattle breeds. Animal.

[45] Lagerstedt A., Lundström K., Lindahl G. (2011). Influence of vacuum or high-oxygen modified atmosphere packaging on quality of beef M. longissimus dorsi steaks after different ageing times. Meat Sci..

[46] Holman B.W.B., van de Ven R.J., Mao Y., Coombs C.E.O., Hopkins D.L. (2017). Using instrumental (CIE and reflectance) measures to predict consumers’ acceptance of beef colour. Meat Sci..

[47] Hernández Salueña B., Sáenz Gamasa C., Diñeiro Rubial J.M., Alberdi Odriozola C. (2019). CIELAB color paths during meat shelf life. Meat Sci..

[48] Hopkins D.L., Ponnampalam E.N., van de Ven R.J., Warner R.D. (2014). The effect of pH decline rate on the meat and eating quality of beef carcasses. Anim. Prod. Sci..

[49] Khliji S., van de Ven R., Lamb T.A., Lanza M., Hopkins D.L. (2010). Relationship between consumer ranking of lamb colour and objective measures of colour. Meat Sci..

